# Effect of Step Gate Work Function on InGaAs p-TFET for Low Power Switching Applications

**DOI:** 10.3390/nano11123166

**Published:** 2021-11-23

**Authors:** Sayed Md Tariful Azam, Abu Saleh Md Bakibillah, Md Tanvir Hasan, Md Abdus Samad Kamal

**Affiliations:** 1 Department of Electrical and Computer Engineering, Technische Universität Kaiserslautern, 67653 Kaiserslautern, Germany; sazam@rhrk.uni-kl.de; 2 Department of Electrical and Electronic Engineering, Jashore University of Science and Technology, Jashore 7408, Bangladesh; tan_vir_bd@yahoo.com; 3 School of Engineering, Monash University, Bandar Sunway, Subang Jaya 47500, Malaysia; 4 Graduate School of Science and Technology, Gunma University, 1-5-1 Tenjincho, Kiryu 376-8515, Japan

**Keywords:** p-TFET, gate work function, dual material gate, InGaAs, low power switching

## Abstract

In this study, we theoretically investigated the effect of step gate work function on the InGaAs p-TFET device, which is formed by dual material gate (DMG). We analyzed the performance parameters of the device for low power digital and analog applications based on the gate work function difference (∆ϕ_S-D_) of the source (ϕ_S_) and drain (ϕ_D_) side gate electrodes. In particular, the work function of the drain (ϕ_D_) side gate electrodes was varied with respect to the high work function of the source side gate electrode (Pt, ϕ_S_ = 5.65 eV) to produce the step gate work function. It was found that the device performance varies with the variation of gate work function difference (∆ϕ_S-D_) due to a change in the electric field distribution, which also changes the carrier (hole) distribution of the device. We achieved low subthreshold slope (SS) and off-state current (I_off_) of 30.89 mV/dec and 0.39 pA/µm, respectively, as well as low power dissipation, when the gate work function difference (∆ϕ_S-D_ = 1.02 eV) was high. Therefore, the device can be a potential candidate for the future low power digital applications. On the other hand, high transconductance (g_m_), high cut-off frequency (f_T_), and low output conductance (g_d_) of the device at low gate work function difference (∆ϕ_S-D_ = 0.61 eV) make it a viable candidate for the future low power analog applications.

## 1. Introduction

The band-to-band tunneling transport mechanism of tunnel field effect transistors (TFETs) allows the device to operate on low supply voltage (V_DD_) and to overcome the subthreshold slope limit (SS ≥ 60 mV/dec) of traditional metal oxide semiconductor field effect transistors (MOSFETs), which makes TFET a potential candidate for the future low power devices [1,2,3,4,5]. TFETs have lower power consumption in digital circuits and have higher sensitivity and transconductance per unit of current in analog circuits compared to the conventional MOSFETs [1,3,6,7,8]. In particular, low and direct bandgap III–V materials have attracted a lot of attention for TFET devices, due to their inherent material properties (such as direct band gap, high electron mobility, and low exciton binding energy) as compared to Si [2]. They have also higher tunneling efficiency due to their shorter tunneling distance and lower phonon emission. Among these materials, ternary III–V materials have a higher degree of compositional dependency, allowing designers to fine-tune the material properties to meet their requirements [9,10]. Moreover, nowadays, InGaAs is a very suitable material for TFET devices leading to open new opportunities to make the compact integrated circuits for next generation electronic as well as optoelectronic/photonic applications [2].

Unlike n-TFETs, p-TFETs (usually n-i-p doping structure) with III–V materials have built-in issues [2]. Due to the low conduction band density of states of III–V materials, a heavily n-doped source of p-TFETs induces large conduction band degeneracy, which causes exponential tail from Fermi distribution and thus, SS is increased. The optimal source doping should be lower than n-TFETs while focused on steep SS. On the other hand, reduced source doping results in a lower electric field at the tunnel junction, which reduces drain current (I_D_). To date, the counter effect of doping on I_D_ and SS has been subsidized by using heterostructure or a heavily counter doped pocket between the source and channel regions to achieve steep SS and high I_D_ with high I_on_/I_off_, similar to n-TFETs (for complementary switching) [10,11,12,13]. In line with this expectation, the dual material gate (DMG) design is a leading contender for achieving steep SS and high I_D_, because it combines the advantages of dual-material-gate and double-gate structures.

The DMG design was first proposed by Wei Long to suppress the short channel effect of MOSFET devices [14], where it was shown that in DMG devices instead of a single metal gate, two metal gates are positioned laterally on the gate region and the gate contact on the source side has higher work function than the gate contact on the drain side. The DMG structure reduces the electric field on the drain region and hence, the electric field is distributed, which increases channel efficiency. The distributed electric field and higher peak on the source side of the channel accelerate the charge carriers more rapidly, which makes DMG devices a potential candidate for high-speed applications. The DMG design is investigated in various recent devices, e.g., a DMG design in CNT-FET is reported in [15], the applicability of DMG devices for digital applications using gate-all-around (GAA) and GaN are reported in [16,17] and the applicability of DMG devices for subthreshold analog/RF applications are reported in [18,19]. The DMG designs are also explored for TFETs [20,21,22,23,24].

However, to the best of our knowledge, no DMG design for the InGaAs p-TFET device has been reported. For this paper, we investigated the DMG design on an InGaAs p-TFET device in terms of the step gate work function produced by the work function difference between the source and drain side gate electrodes. The work function of the drain side gate electrodes was regulated with reference to the high work function of the source side gate electrodes to generate the step gate work function. Our approach of using dual material gate with different work functions was inspired by our previous work [17], where the performance of sub-10-nm GaN-based DG-MOSFETs with different gate work function combinations were investigated and it was found that the short-channel effects (SCEs) can be significantly reduced using gates made of dual materials. We inspected the device’s suitability for low-power digital and analog applications by analyzing capacitance and performance parameters. The results show improvements in I_on_, I_off_, I_on_/I_off_, SS, and DIBL over the reported data in this domain [23]. The paper is organized as follows. Section 2 describes the device structure and Section 3 provides the computation methods. Section 4 presents the results of transfer characteristics, output characteristics, and physical properties of the device. Section 5 gives the capacitance characteristics of the device and device level performance parameters for low power digital and analog applications. Finally, Section 6 draws the conclusion.

## 2. Device Structure

**Figure 1:**
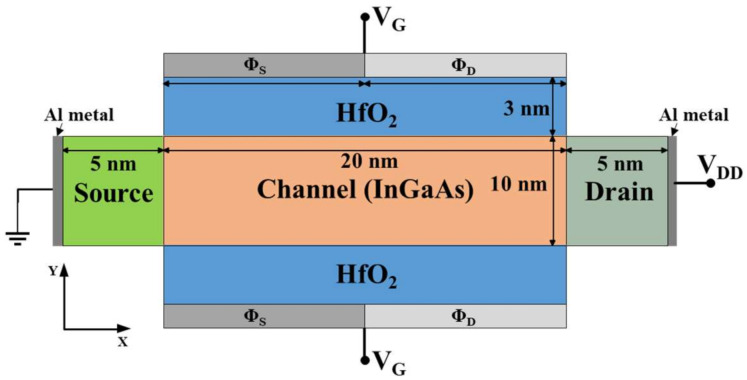
Schematic structure of the proposed double gate p-InGaAs TFET device.

In this paper, a double gate p-TFET device has been studied using dual material gate structure to improve the device performance. The structure of the proposed p-type InGaAs DMG-TFET device is illustrated in Figure 1, where the source, channel, and drain lengths are 5 nm, 20 nm, and 5 nm, respectively [5]. We considered gate width as 1 µm. For the proposed device, a channel thickness of 10 nm and a physical gate oxide (HfO_2_, ε = 22 ε_0_) thickness of 3 nm were used. Our study mainly exploited a 2D simulation setup with cross-sectional view of the proposed p-TFET device structure, where *x*- and *y*-axes are defined along the channel length and channel thickness, respectively. The doping concentrations, i.e., acceptor (N_A_) and donor (N_D_) of source and drain regions were considered as 1 × 10^19^ cm^−3^ (N_D_) and 5 × 10^18^ cm^−3^ (N_A_), respectively. In the channel region light, doping concentration of 1 × 10^16^ cm^−3^ (N_D_) was used.

The formation of step gate work function requires two types of gate electrodes, e.g., high and low work function electrodes on the source side (ϕ_S_) and the drain side (ϕ_D_), respectively. In this work, we considered the same length for both electrodes, i.e., L_ϕS_ = L_ϕD_ = 10 nm. Since a high work function source side gate electrode improves carrier efficiency in channel under the ϕ_S_ region [20,24], we considered ϕ_S_ = 5.65 eV (Pt). On the other hand, low work function gate electrodes such as Ni, Mo, and W were employed in the ϕ_D_ region. In the literature, the gate electrodes on the source are denoted as the tunneling (control) and auxiliary (screen) gates, respectively [22,23,24]. The device performance is analyzed in terms of step gate work function induced by the difference in work function (∆ϕ_S-D_) between the source side (ϕ_S_) and the drain side (ϕ_D_) gate electrodes. The differences in work function (∆ϕ_S-D_) considered in this study are given in Table 1.

## 3. Computational Methods

We conducted all simulations using Silvaco ATLAS TCAD [25] and the simulation setup was adopted from our previous work [5] and Kim et al. [9]. The carrier distribution was calculated using the Fermi model. To compute the carrier recombination, we used the Shockley–Read–Hall (SRH) and auger recombination models, as well as the bandgap narrowing model that describes the high doping effect on the bandgap. Low field mobility due to doping density was taken into account by the concentration dependent mobility model, while field velocity saturation was taken into account by the field dependent mobility model. We considered quantum effects using the density gradient quantum moments model [5,9]. To tunnel through the bandgap using trap states, we used the trap assisted tunneling model with phonon scattering effect. A nonlocal band-to-band tunneling model was used to explain nonlocal interband tunneling effect. The on-state (source-to-channel) and off-state (drain-to-channel) tunneling were considered as separate tunneling regions. The tunneling probability *T*(*E*) is calculated as

(1) TE =exp−42m∗Eg323eℏξ

where $m^{*}$ is the effective mass, $E_{g}$ is the bandgap energy, ξ is the electric field, and ℏ is the reduced Planck constant. The simulations were performed at room temperature (300 K). In this paper, I_on_ and I_off_ are considered as drain current (I_D_) at V_DD_ = V_GS_ = −0.5 V and V_DD_ = −0.5 V, V_GS_ = 0 V, respectively. The DIBL and SS were calculated at constant I_D_ of 1 × 10^−9^ A/µm. The simulations were also carried out at a frequency of 1 MHz.

## 4. Results and Discussion

**Figure 2:**
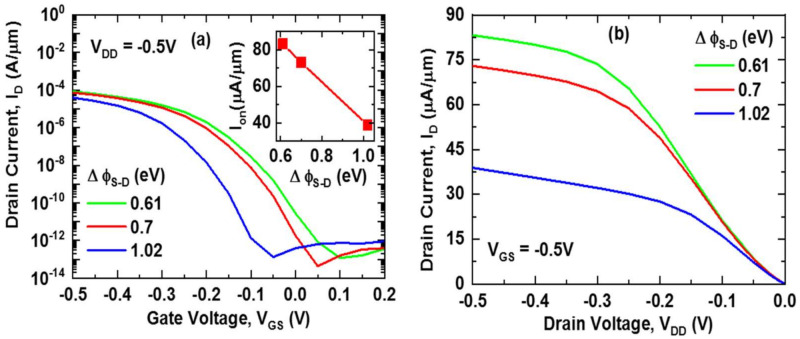
(**a**) Transfer characteristics (I_D_-V_GS_) at V_DD_ = −0.5 V; inset shows I_on_ as a function of work function difference (∆ϕ_S-D_) and (**b**) output characteristics (I_D_-V_DD_) at V_GS_ = −0.5 V.

The transfer characteristics of the device for differences in work functions are shown in Figure 2a. It is found that for the lowest work function difference (∆ϕ_S-D_ = 0.61 eV), the device exhibits the highest I_on_ (83.2 µA/µm) and I_off_ (28.3 pA/µm). The inset figure of Figure 2a shows that I_on_ decreases linearly from 83.2 µA/µm to 38.9 µA/µm when the work function difference is increased from ∆ϕ_S-D_ = 0.61 eV to ∆ϕ_S-D_ = 1.02 eV. Figure 2b shows the output characteristics of the device at V_GS_ = −0.5 V, where the I_D_-V_DD_ curve shows that the increasing rate of drain current (I_D_) with respect to drain voltage (V_DD_) is higher for ∆ϕ_S-D_ = 0.61 eV and ∆ϕ_S-D_ = 0.7 eV compared to ∆ϕ_S-D_ = 1.02 eV, which means that saturation is not reached yet for ∆ϕ_S-D_ = 1.02 eV. Delayed saturation characteristics of ∆ϕ_S-D_ = 1.02 eV can be improved by higher source doping that reduces the source depletion [26]. On the other hand, higher source doping increases SS of the device and introduces Fermi tail.

**Figure 3:**
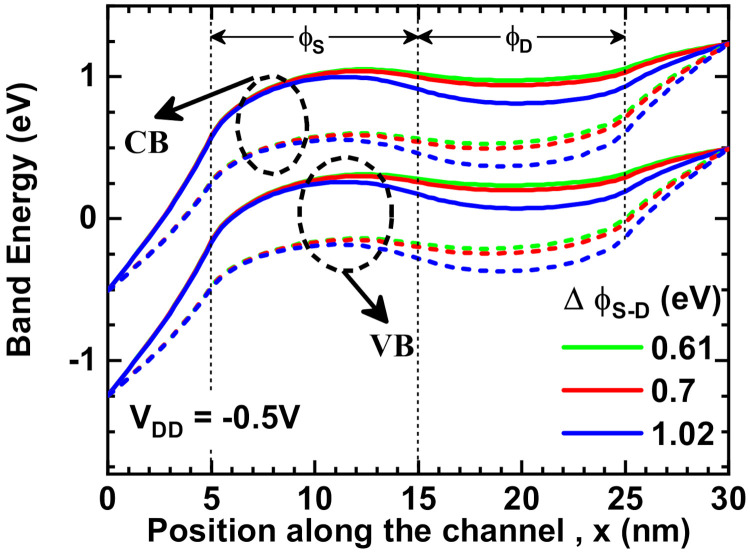
Conduction band (CB) and valance band (VB) profiles along the channel—dot line: off-state (V_DD_ = −0.5 V, V_GS_ = 0 V), solid line: on-state (V_DD_ = V_GS_ = −0.5 V).

The band profiles of the device for both off-state (V_DD_ = −0.5 V, V_GS_ = 0 V) and on-state (V_DD_ = V_GS_ = −0.5 V) are shown in Figure 3. In a p-TFET, the on-state negative gate bias shifts the bands up to align the conduction band of the source region with the valence band of the channel region, allowing holes to tunnel from the conduction band to the valence band. In other words, the electron tunnels from the valence band of the channel region to the conduction band of the source region. In this condition, the potential of the high work function gate electrode is higher than the potential of the low work function gate electrode for the same applied negative gate bias and the effect is reflected in the channel region. It is found that in the ϕ_S_ region, a high work function gate electrode Pt causes both the conduction band and the valence band to have a high potential in all ∆ϕ_S-D_ conditions. On the contrary, both the conduction band and the valence band have lower potential in the ϕ_D_ region (under the low work function electrode) than in the ϕ_S_ region, and their potential varies according to the work function of the gate electrodes. As a result, the energy bands of device in the channel region show step-like (or undulated) features. Furthermore, a lower potential in the ϕ_D_ region indicates that carriers in that region have less energy. Hence, the drain to channel tunneling probability is low in the off-state.

**Figure 4:**
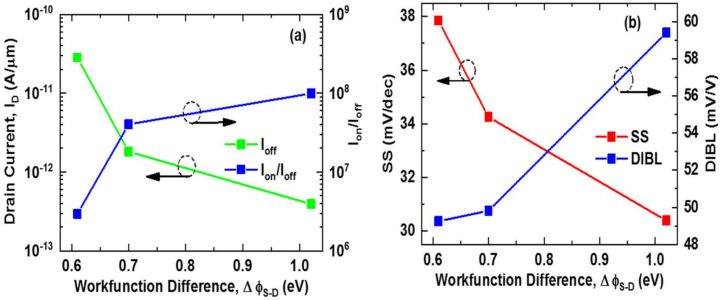
(**a**) I_off_ and I_on_/I_off_ and (**b**) SS and DIBL as a function of work function difference (∆ϕ_S-D_).

The fluctuation of I_off_, I_on_/I_off_, SS, and DIBL as a function of gate work function difference (∆ϕ_S-D_) is shown in Figure 4a,b. When the work function difference is increased from 0.61 eV to 1.02 eV, I_off_ reduces on logarithmic scale from 28.3 pA/m to 0.39 pA/m. As a result, at ∆ϕ_S-D_ = 1.02 eV, the highest I_on_/I_off_ ratio of 9.94 × 10^7^ is obtained. It is also observed that SS drops with an increase in ∆ϕ_S-D_. The lowest SS of 30.89 mV/dec is achieved for ∆ϕ_S-D_ = 1.02 eV. The highest SS of 37.84 mV/dec is observed when ∆ϕ_S-D_ = 0.61 eV, which is still less than the traditional SS limit of 60 mV/dec. Unlike SS, the DIBL of the device increases with the increase of ∆ϕ_S-D_. We found approximately the same DIBL for ∆ϕ_S-D_ = 0.61 eV and ∆ϕ_S-D_ = 0.7 eV, which are 49.25 mV/V and 49.81 mV/V, respectively. In the case of ∆ϕ_S-D_ = 1.02 eV, the highest DIBL of 59.41 mV/V is achieved.

**Figure 5:**
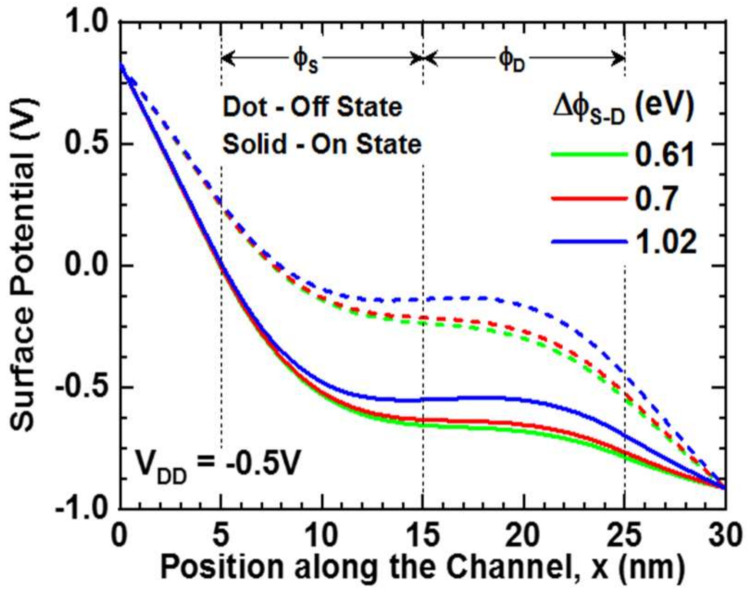
Surface Potential along the channel—dot line: off-state (V_DD_ = −0.5 V, V_GS_ = 0 V), solid line: on-state (V_DD_ = V_GS_ = −0.5 V).

The surface potential of the device in the off-state (V_DD_ = −0.5 V, V_GS_ = 0 V) and on-state (V_DD_ = V_GS_ = −0.5 V) is shown in Figure 5. The negative drain bias and gate bias reduce the surface potential in the drain and channel regions, respectively, while the surface potential in the grounded source region remains higher. However, in the channel region, the surface potential varies according to the work function difference (∆ϕ_S-D_). Hence, the highest and lowest surface potentials in the channel region, respectively, are caused by high work function difference (∆ϕ_S-D_ = 1.02 eV) and low work function difference (∆ϕ_S-D_ = 0.61 eV). The surface potential of single material gate devices remains constant through the channel region according to their work function. However, the surface potential of dual material gate devices introduces a step-like feature in the channel region according to their respective work functions [16,23].

As shown in Figure 5, a high work function electrode has a strong impact in the ϕ_S_ region. As a result, the surface potential dips in the middle of the ϕ_S_ region, forming a trough. On the other hand, a low work function in the ϕ_D_ region increases surface potential in the middle of ϕ_D_ region, creating a crest that shades the ϕ_S_ region from the high drain bias (V_DD_) effect. As a result, the surface potential difference between the ϕ_S_ and ϕ_D_ regions forms the step-like feature in the channel region. When ∆ϕ_S-D_ = 0.61 eV and 0.7 eV, the surface potential in the crest is lower than the trough surface potential; therefore, it is approximately constant (small gradient) around the metal junction. However, when ∆ϕ_S-D_ = 1.02 eV, the crest and trough surface potentials are approximately the same, and the surface potential becomes constant (flat) around the metal junction.

**Figure 6:**
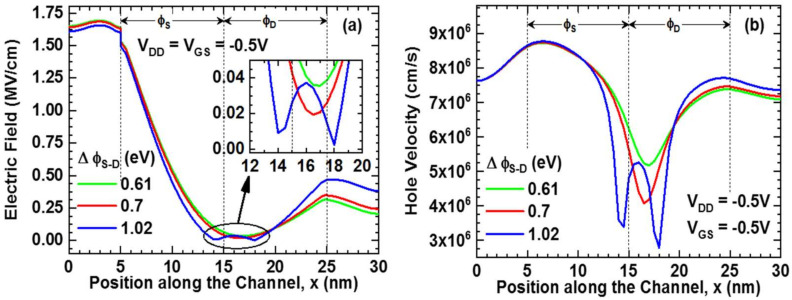
(**a**) Electric Field; inset shows zoomed electric field at the circled region and (**b**) Hole velocity along the channel at on-state (V_DD_ = V_GS_ = −0.5 V).

Figure 6a,b show the electric field and hole velocity of the device, respectively. The high electric field in the tunnel junction of the source and channel region is nearly the same in all circumstances due to the same ϕ_S_ electrode. The electric field fluctuates with ϕ_D_ in the drain region, with the largest peak occurring at the channel–drain junction for a high work function difference (∆ϕ_S-D_ = 1.02 eV). The work function difference between two electrodes raises negative peaks around the junction [23,24], as shown in the inset of the figure. It is found that a low work function difference (∆ϕ_S-D_ = 0.61 eV) has the lowest negative peak. On the contrary, a high work function difference (∆ϕ_S-D_ = 1.02 eV) has the two highest negative peaks. The opposite potential trend appears at the transition of the two gates is responsible for negative electric field peaks [24]. Since the carrier velocity (here, majority carriers are holes) are proportional to the electric field, in Figure 6b, the hole velocity imitates the peaks of the electric field curves. Like the electric field, hole velocity is high at the source–channel junction and nearly constant in all circumstances. The hole velocity drops around the junction of two electrodes and has negative peaks. Then, the velocity increases again towards the drain.

**Figure 7:**
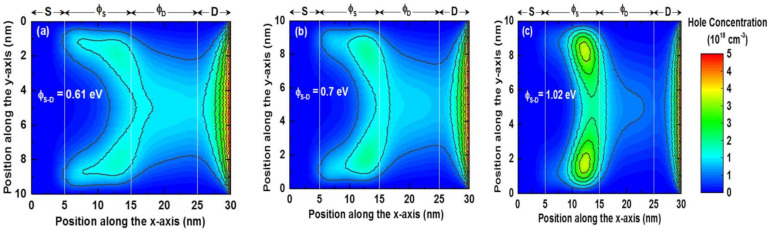
Hole concentration contours at on-state (V_DD_ = V_GS_ = −0.5 V) for (**a**) ∆ϕ_S-D_ = 0.61 eV, (**b**) ∆ϕ_S-D_ = 0.7 eV, and (**c**) ∆ϕ_S-D_ = 1.02 eV.

The on-state hole concentration contour plots of the device for different cases are shown in Figure 7. In Figure 7a, the hole concentration in the ϕ_S_ region is higher than the ϕ_D_ region for ∆ϕ_S-D_ = 0.61 eV. In Figure 7b, the hole concentration becomes confined in the ϕ_S_ region for ∆ϕ_S-D_ = 0.7 eV. It is found that the hole concentration in the ϕ_D_ and drain regions are lower than the previous case. In these two cases, holes are distributed from the ϕ_S_ region to the drain region. However, in Figure 7c, holes are more confined in the ϕ_S_ region near the metal junction, and poorly distributed in the ϕ_D_ and drain regions when ∆ϕ_S-D_ = 1.02 eV. Note that, in all cases, the hole concentration in the ϕ_S_ region is higher on semiconductor–dielectric material interface than the middle of the channel (along *y*-axis). On the contrary, in the ϕ_D_ region, holes are only distributed in the middle of the channel (along *y*-axis). When compared to the electric fields of the device in Figure 6a, it appears that the electric field decreases as hole confinement increases in the ϕ_S_ region. The device’s performance parameters for digital and analog applications are examined in the next section.

## 5. Performance Parameter Analysis

### 5.1. Capacitance Analysis

**Figure 8:**
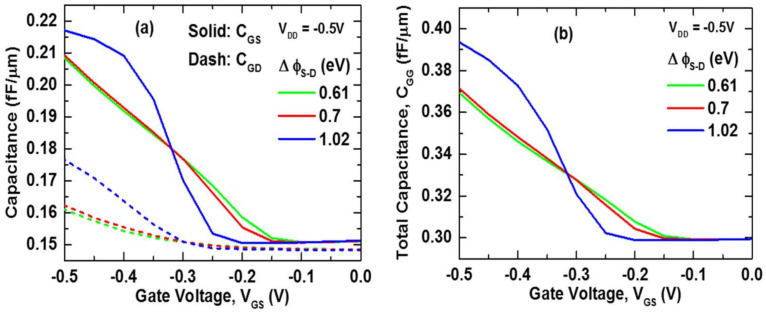
(**a**) Dot line: Gate-to-Drain (C_GD_) and solid line: Gate-to-Source (C_GS_) capacitance (**b**) total capacitance (C_GG_) as a function of Gate Voltage (V_GS_) at V_DD_ = −0.5 V.

We started by examining the device’s C-V curves for different gate work functions (as shown in Figure 8), as capacitance characteristics are crucial in analyzing both digital and analog device performance. In Figure 8a, a high work function electrode (Pt) on the ϕ_S_ region results in a high gate-to-source (C_GS_) capacitance. On the other hand, low work function materials on the ϕ_D_ region reduce gate-to-drain (C_GD_) capacitances in all circumstances. Hence, we achieved a negligible miller effect, which has been a significant concern for TFET devices [27], and reduced output voltage overshoot and undershoot is expected in large-signal transient response in all circumstances. Moreover, Figure 8a shows that for ∆ϕ_S-D_ = 1.02 eV, both gate-to-source (C_GS_) and gate-to-drain (C_GD_) capacitances are higher than the other two cases. Figure 8b depicts the total (gate) capacitance (C_GG_~C_GS_ + C_GD_), which exhibits the similar characteristics, with the highest and lowest total (gate) capacitance (C_GG_) being ∆ϕ_S-D_ = 1.02 eV and ∆ϕ_S-D_ = 0.61 eV, respectively. The total (gate) capacitance (C_GG_) remains approximately the same when ∆ϕ_S-D_ = 0.61 eV and ∆ϕ_S-D_ = 0.7 eV.

### 5.2. Digital Performance Parameters

To investigate the device’s digital performance, different parameters were considered, e.g., intrinsic speed (τ = C_GG_V_DD_/I_on_), leakage power (P_leak_ = nI_off_V_DD_), dynamic power (P_dyn_ = 0.5 × nI_on_V_DD_α), total power (P_total_ = P_leak_ + P_dyn_), dynamic energy (E_dyn_ = 0.5 × nC_GG_V^2^_DD_α), leakage energy (E_leak_ = n^2^I_off_V_DD_τ), and total energy (E_total_ = E_leak_ + E_dyn_). The logic depth n = 50 and activity factor α = 2% were used [28]. The calculated values of these parameters are listed in Table 2.

It is found that the highest gate capacitance (C_GG_) at ∆ϕ_S-D_ = 1.02 eV results in ~2 times higher intrinsic speed (τ). The leakage power (P_leak_) is proportional to I_off_ and therefore, P_leak_ dissipation at ∆ϕ_S-D_ = 1.02 eV is ~13.78 × 10^−3^ times lower than at ∆ϕ_S-D_ = 0.61 eV. In addition, the lowest I_on_ at ∆ϕ_S-D_ = 1.02 eV results in the lowest dynamic power (P_dyn_). Therefore, ∆ϕ_S-D_ = 1.02 eV has the lowest total power (P_total_) dissipation. A high gate capacitance (C_GG_) of ∆ϕ_S-D_ = 1.02 eV slightly increases the dynamic energy consumption (E_dyn_) of the device than other cases of ∆ϕ_S-D_. In spite of the highest intrinsic speed (τ) at ∆ϕ_S-D_ = 1.02 eV, the lowest I_off_ of the device gives the lowest leakage energy consumption (E_leak_). The result shows that the dynamic component of power and energy of the device play the key role in total power (P_total_) dissipation and energy (E_total_) consumption of the device for different ∆ϕ_S-D_. Since the low power device is the primary concern for modern digital applications, with the lowest power dissipation at ∆ϕ_S-D_ = 1.02 eV (about half of the other two cases and slightly higher energy consumption), the high work function difference model fits well for digital application requirements.

### 5.3. RF Performance Parameters

**Figure 9:**
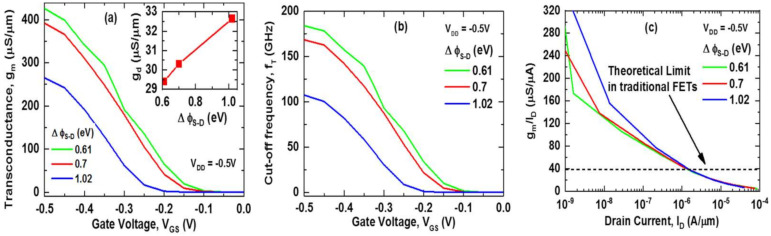
(**a**) Transconductance (g_m_), (**b**) cut-off frequency (f_T_) as function of Gate Voltage (V_GS_), and (**c**) transconductance to current ratio (gm/Id) as function of drain current (Id) at V_DD_ = −0.5 V; inset shows output conductance (g_d_) at V_DD_ = V_GS_ = −0.5 V as a function of work function difference (∆ϕ_S-D_).

The device’s RF performance was measured in terms of transconductance (g_m_ = I_D_/V_GS_), output conductance (g_d_ = dI_D_/dV_DD_), cut-off frequency (f_T_ = g_m_/2πC_GG_), and transconductance generation factor (TGF = g_m_/I_D_). Figure 9a,b shows the transconductance (g_m_) and cut-off frequency (f_T_) of the device for different ∆ϕ_S-D_ as a function of gate voltage (V_GS_). In Figure 9a, the inset figure shows the output conductance (g_d_) as a function of ∆ϕ_S-D_ at V_GS_ = −0.5 V. High transconductance (g_m_) ensures high amplification and high cut-off frequency (f_T_) is the key parameter for high-speed applications to analyze the device’s gain [29]. The highest drain current (I_D_) at ∆ϕ_S-D_ = 0.61 eV results in the highest transconductance g_m_ (426.29 µS/µm), which is ~1.605 times higher than ∆ϕ_S-D_ = 1.02 eV. The lowest output conductance (g_d_) and highest transconductance (g_m_) of ∆ϕ_S-D_ = 0.61 eV result in the highest voltage gain (A_v_ = g_m_/g_d_), as seen in the inset of Figure 9a. The lowest gate capacitance (C_GG_) and highest transconductance of the device at ∆ϕ_S-D_ = 0.61 eV also gives the highest cut-off frequency f_T_ = 183.72 GHz, which is 1.71 times higher than at ∆ϕ_S-D_ = 1.02 eV. Figure 9c depicts the device’s transconductance generation factor (TGF) as a function of drain current (I_D_), which is a key parameter for low power analog subthreshold applications [30]. It is referred to as the transconductance-to-current ratio (g_m_/I_d_) as well as device efficiency in the literature [29]. It measures the efficiency of the device to convert current (power) into transconductance (speed) [31]. Due to steep SS of the device at ∆ϕ_S-D_ = 1.02 eV, the TGF is also found to be steep. Here, all cases match the traditional FET limit (38.5 µS/µA) for the same drain current (I_D_). In the subthreshold region, ∆ϕ_S-D_ = 0.61 eV has higher TGF than ∆ϕ_S-D_ = 0.7 eV and lower TGF than ∆ϕ_S-D_ = 1.02 eV. In capacitive load circuits, a lower TGF implies lower input drivability, which indicates higher power dissipation. On the other hand, higher TGF costs in terms of linearity of the device [30]. With the highest transconductance (g_m_), voltage gain (A_v_), and cut-off frequency (f_T_), the device at ∆ϕ_S-D_ = 0.61 eV can be a suitable candidate for future low-power analog applications.

## 6. Conclusions

In this paper, we have investigated the effect of step gate work function on the InGaAs p-TFET device based on the gate work function difference (∆ϕ_S-D_) of the source (ϕ_S_) and drain (ϕ_D_) side gate electrodes. Firstly, we have analyzed the transfer and output characteristics, and physical properties of the device. The results show that the work function difference (∆ϕ_S-D_) changes the electric field on the channel by creating a potential difference in energy bands between the ϕ_S_ and ϕ_D_ regions. We achieved the lowest SS (30.86 mV/dec), I_off_ (0.39 × 10^−12^ A/µm), and minimum I_on_/I_off_ ratio (9.97 × 10^7^) for high work function difference ∆ϕ_S-D_ = 1.02 eV. Finally, we have explored the digital and analog performance parameters at the device level. It is found that the device with a high work function difference ∆ϕ_S-D_ = 1.02 eV dissipates the least amount of power and consumes the least amount of leakage energy, making it suitable for digital applications. On the other hand, a low work function difference ∆ϕ_S-D_ = 0.61 eV results in the lowest output conductance g_d_ (29.4 µS/µm), maximum transconductance g_m_ (426 µS/µm), and cut-off frequency f_T_ (184 GH_z_). At ∆ϕ_S-D_ = 0.61 eV, the lowest g_d_ and highest g_m_ produce the largest voltage gain, which is an important parameter in analog applications. The findings reveal that the InGaAs p-TFET can be used for both low-power digital and analog applications by tuning the step gate work function of the device. From the above discussion, we can conclude that InGaAs TFETs will be suitable for future low-power integrated switching circuit applications.

## Figures and Tables

**Table 1:** Work function combinations and differences considered in this study.

Source Side Electrode, ϕ_S_ (eV)	Drain Side Electrode, ϕ_D_ (eV)	Work Function Difference, ∆ϕ_S-D_ (eV)
Pt (5.65)	Ni (5.04)	0.61
	Mo (4.95)	0.70
	W (4.63)	1.02

**Table 2:** Digital performance parameters.

		∆ϕ_S-D_ (eV)	
**Parameters**	**0.61**	**0.7**	**1.02**
τ (ps)	2.22	2.55	5.07
P_leak_ (µW/µm)	7.09 × 10^−4^	4.53 × 10^−5^	9.77 × 10^−6^
P_dyn_ (µW/µm)	20.8	18.2	9.71
P_total_ (µW/µm)	20.8	18.2	9.71
E_leak_ (aJ/µm)	78.6 × 10^−3^	5.77 × 10^−3^	2.47 × 10^−3^
E_dyn_ (aJ/µm)	46.2	46.4	49.2
E_total_ (aJ/µm)	46.2	46.4	49.2

## Data Availability

Not applicable.

## References

[1] Datta S., Bijesh R., Liu H., Mohata D., Narayanan V. (2013). Tunnel transistors for energy efficient computing. IEEE Int. Reliab. Phys. Symp..

[2] Lu H., Seabaugh A. (2014). Tunnel field-effect transistors: State-of-the-art. IEEE J. Electron Devices Soc..

[3] Datta S., Liu H., Narayanan V. (2014). Tunnel FET technology: A reliability perspective. Microelectron. Reliab..

[4] Fischer I.A., Bakibillah A.S.M., Golve M., Hahnel D., Isemann H., Kottantharayil A., Oehme M., Schulze J. (2012). Silicon tunneling field-effect transistors with tunneling in line with the gate field. IEEE Electron Device Lett..

[5] Azam S.M.T., Bakibillah A.S.M., Kamal M.A.S. (2019). Performance Evaluation of InGaAs Dielectric Engineered Tunnel Field-Effect Transistors. J. Nano Res..

[6] Sedighi B., Hu X.S., Liu H., Nahas J.J., Niemier M. (2014). Analog Circuit Design Using Tunnel-FETs. IEEE Trans. Circuits Syst. I Regul. Pap..

[7] Biswas A., Luong G.V., Chowdhury M.F., Alper C., Zhao Q.T., Udrea F., Mantl S., Ionescu A.M. (2017). Benchmarking of Homojunction Strained-Si NW Tunnel FETs for Basic Analog Functions. IEEE Trans. Electron Devices.

[8] Settino F., Lanuzza M., Strangio S., Crupi F., Palestri P., Esseni D., Selmi L. (2017). Understanding the potential and limitations of tunnel FETs for low-voltage analog/mixed-signal circuits. IEEE Trans. Electron Devices.

[9] Kim Y.J., Yoon Y.J., Seo J.H., Lee S.M., Cho S., Lee J.H., Kang I.M. (2014). Effect of Ga fraction in InGaAs channel on performances of gate-all-around tunneling field-effect transistor. Semicond. Sci. Technol..

[10] Knoch J., Appenzeller J. (2010). Modeling of high-performance p-type III-V heterojunction tunnel FETs. IEEE Electron Device Lett..

[11] Verhulst A.S., Verreck D., Pourghaderi M.A., Van de Put M., Soree B., Groeseneken G., Collaert N., Thean A.Y. (2014). Can p-channel tunnel field-effect transistors perform as good as n-channel?. Appl. Phys. Lett..

[12] Verreck D., Verhulst A.S., Sorée B., Collaert N., Mocuta A., Thean A., Groeseneken G. (2014). Improved source design for p-type tunnel field-effect transistors: Towards truly complementary logic. Appl. Phys. Lett..

[13] Huang J.Z., Long P., Povolotskyi M., Klimeck G., Rodwell M.J. (2016). P-type tunnel FETs with triple heterojunctions. IEEE J. Electron Devices Soc..

[14] Long W., Ou H., Kuo J.M., Chin K.K. (1999). Dual-material gate (DMG) field effect transistor. IEEE Trans. Electron Devices.

[15] Orouji A.A., Arefinia Z. (2009). Detailed simulation study of a dual material gate carbon nanotube field-effect transistor. Phys. E Low-Dimens. Syst. Nanostruct..

[16] Djeffal F., Lakhdar N., Yousfi A. (2011). An optimized design of 10-nm-scale dual-material surrounded gate MOSFETs for digital circuit applications. Phys. E Low-Dimens. Syst. Nanostruct..

[17] Mehedi I.M., Alshareef A.M., Islam M.R., Hasan M.T. (2018). GaN-based double-gate (DG) sub-10-nm MOSFETs: Effects of gate work function. J. Comput. Electron..

[18] Chakraborty S., Mallik A., Sarkar C.K. (2008). Subthreshold performance of dual-material gate CMOS devices and circuits for ultralow power analog/mixed-signal applications. IEEE Trans. Electron Devices.

[19] Kundu A., Koley K., Dutta A., Sarkar C.K. (2014). Impact of gate metal work-function engineering for enhancement of subthreshold analog/RF performance of underlap dual material gate DG-FET. Microelectron. Reliab..

[20] Saurabh S., Kumar M.J. (2010). Novel attributes of a dual material gate nanoscale tunnel field-effect transistor. IEEE Trans. Electron Devices.

[21] Lv Y., Huang Q., Wang H., Chang S., He J. (2016). A numerical study on graphene nanoribbon heterojunction dual-material gate tunnel FET. IEEE Electron Device Lett..

[22] Noor S.L., Safa S., Khan M.Z.R. (2016). Dual-material double-gate tunnel FET: Gate threshold voltage modeling and extraction. J. Comput. Electron..

[23] Vishnoi R., Kumar M.J. (2014). Compact analytical model of dual material gate tunneling field-effect transistor using interband tunneling and channel transport. IEEE Trans. Electron Devices.

[24] Zhang A., Mei J., Zhang L., He H., He J., Chan M. Numerical study on dual material gate nanowire tunnel field-effect transistor. Proceedings of the IEEE International Conference on Electron Devices and Solid-State Circuit (EDSSC).

[25] SILVACO (2014). ATLAS User’s Manual.

[26] Rajamohanan B., Mohata D., Ali A., Datta S. (2013). Insight into the output characteristics of III-V tunneling field effect transistors. Appl. Phys. Lett..

[27] Mookerjea S., Krishnan R., Datta S., Narayanan V. (2009). On enhanced Miller capacitance effect in interband tunnel transistors. IEEE Electron Device Lett..

[28] Zhang Q., Seabaugh A. Can the interband tunnel FET outperform Si CMOS?. Proceedings of the IEEE Device Research Conference.

[29] Kondekar P.N., Nigam K., Pandey S., Sharma D. (2016). Design and Analysis of Polarity Controlled Electrically Doped Tunnel FET With Bandgap Engineering for Analog/RF Applications. IEEE Trans. Electron Devices.

[30] Sarkar A., Das A.K., De S., Sarkar C.K. (2012). Effect of gate engineering in double-gate MOSFETs for analog/RF applications. Microelectron. J..

[31] Madan J., Chaujar R. (2016). Interfacial charge analysis of heterogeneous gate dielectric-gate all around-tunnel FET for improved device reliability. IEEE Trans. Device Mater. Reliab..

